# Murine-related helminthiasis: a public health concern at solid waste sites around forest- adjacent communities in Thailand

**DOI:** 10.3389/fvets.2024.1463046

**Published:** 2025-01-15

**Authors:** Nattapon Maneepairoj, Paisin Lekcharoen, Kittipong Chaisiri, Supaphen Sripiboon

**Affiliations:** ^1^Department of Large Animal and Wildlife Clinical Sciences, Faculty of Veterinary Medicine, Kasetsart University, Bangkok, Thailand; ^2^Department of Veterinary Public Health, Faculty of Veterinary Science, Chulalongkorn University, Bangkok, Thailand; ^3^Department of Helminthology, Faculty of Tropical Medicine, Mahidol University, Bangkok, Thailand

**Keywords:** helminth, helminthiasis, murine rodent, solid waste site, Thailand, zoonotic helminth

## Abstract

Murine-related helminthiasis is a frequently overlooked zoonotic disease with significant public health implications. The role of murine rodents in transmitting these infections to other animals remains under-researched. This study aimed to investigate murine-related helminth infections at solid waste sites, particularly in forest-adjacent communities where murine rodent populations are high and multi-host interactions are possible. During a 5-day trapping session, 36 live traps were deployed across different habitats during both wet and dry seasons. Trapped murine rodents and their gastrointestinal (GI) parasites were morphologically evaluated for species identification. The results revealed that a total of 380 murine rodents were captured, with an overall GI helminth infection prevalence of 86.8% (330/380). The adult male murine rodents exhibited higher prevalence, abundance, and species richness of helminths compared to juvenile and female murine rodents. A total of 16 helminth species were identified, with *Trichostrongylus* morphotype A showing the highest infection prevalence (53.2%). Six zoonotic species were also detected, including *Syphacia obvelata* (22.4%), *Syphacia muris* (12.4%), *Raillietina* spp. (10.8%), *Hymenolepis diminuta* (10.3%), *Vampirolepis nana* (10%), and *Cyclodontostomum purvisi* (2.4%). Increased population of murine rodents was observed at the solid waste sites, as indicated by higher trap success (TS) rates. Forest murine rodents exhibited a significant prevalence of helminth infections and high species diversity. These findings suggest that solid waste sites adjacent to forests may pose a heightened risk for disease transmission, warranting further attention.

## Introduction

The exponential increase in global municipal solid waste generation, projected to reach 3.40 billion tons by 2050 (1), poses significant threats to public health and environmental sustainability. The World Health Organization (WHO) emphasizes the complex relationship between poor waste management and the contamination of soil, water, and air (2), which creates substantial health hazards for communities (3, 4). Moreover, inadequate waste management transforms solid waste sites into foraging grounds for a diverse range of animals, including humans. This phenomenon disrupts natural movement patterns and fosters interspecies interactions, potentially increasing the transmission of diseases (4, 5). The cohabitation of diverse species within these environments creates optimal conditions for zoonotic diseases such as leptospirosis, rabies, dengue, and influenza (6), highlighting the urgent need for comprehensive waste management strategies.

Murine rodents (family *Muridae*), including rats and mice, thrive in diverse habitats, particularly human-modified environments such as solid waste sites (7, 8). Murine rodents are not only considered agricultural pests but also serve as reservoirs for numerous zoonotic diseases, including leptospirosis, hantavirus, and several parasitic infections (9–18). Waste sites, with their abundant food resources, may contribute to increased populations of murine rodent and amplify the risk of disease transmission. Although zoonotic diseases in murine rodents have been extensively studied in agricultural and community settings, research specific to solid waste sites is lacking. Addressing this gap is crucial not only for public health but also for mitigating disease transmission to other areas and species (19, 20).

In Thailand, murine rodents are widespread in urban and rural areas, serving as reservoirs for numerous pathogens. Studies have identified murine rodents positive for various microparasites such as *Leptospira* spp., *Orientia* spp., *Bartonella* spp., Hantavirus, Herpes virus, lymphocytic choriomeningitis virus (LCMV), Rabies virus, *Toxoplasma gondii*, *Trypanosoma* spp., and *Babesia* spp. (21–23). Studies on macroparasites have documented ectoparasites such as mites (e.g., *Leptotrombidium* spp. and *Blankaartia* spp.), ticks (e.g., *Dermacentor* spp., *Haemaphysalis* spp., *Ixodes glanulatus,* and *Rhipicephalus sanguieus*), and fleas (e.g., *Xenopsylla cheopis* and *Nosopsyllus fasciatus*). Parasitic nematodes (e.g., *Angiostrongylus cantonensis*, *Calodium hepatium*, *Cyclodontostomum purvisi*, and *Trichuris muris*), cestodes (e.g., *Raillietina* spp., *Hymenolepis diminuta*, *Vampirolepis nana* (syn. *Hymenolepis nana*), and *Hydratigera taeniaeformis*), and trematodes (e.g., *Echinostoma malayanum*) have also been reported (23–33). However, there remains a notable gap in research on gastrointestinal (GI) helminths in murine populations specifically within solid waste sites. These sites could serve as hotspots for parasitic transmission, posing significant risks to public health and ecosystem integrity.

As outlined earlier, solid waste sites represent unique habitat where human activities alter ecological dynamics, including zoonotic disease transmission. Murine rodents frequently inhabit these sites, interacting with multiple species and environmental pathogens. Given their adaptability and close association with human settlements, murine rodents are of particular interest as potential reservoirs of zoonotic diseases. This study aims to fill this gap by investigating the role of murine rodents as potential reservoirs for GI helminths, comparing their abundance and diversity between waste sites and other habitats. In addition, seasonal variations in GI helminth prevalence are explored to better understand parasitic transmission in these understudied environments.

## Materials and methods

### Study sites and sampling locations

To investigate the abundance and diversity of murine hosts and their GI helminths, three study sites were selected in Nakhon Ratchasima Province, Thailand: Soengsang District (*S1*; 14.3593, 102.4172), Khonburi District (*S2*; 14.4651, 102.1621), and Wangnamkhieo District (*S3*; 14.4372, 101.8155). All three study sites (S1-S3) are situated near the Dong Phayayen–Khao Yai Forest Complex. These study sites encompass four distinct habitat types: (1) solid waste sites (SWS); (2) natural forests (NF), including either dipterocarps or secondary forests; (3) dense understory lands (DUL), characterized by abundant and tightly packed vegetation in the understory, creating a dense cover that provides ideal concealment for small mammals (e.g., corn and cassava crop); and (4) sparse understory lands (SUL), characterized by reduced vegetation density in the understory, offering a less extensive cover (e.g., perennial crop and orchards). Each study site contained an SWS and the other three habitats, which were located within a 2×2 km-square area. This study conducted in 3 study sites, in each sites we selected 5 habitats (from any of 4 types of habitats - SWS, NF, DUL, SUL). A map of the sampling locations is shown in Supplementary Figure S1.

### Sampling strategies

During the period 2022–2023, a 5-day trapping session was conducted biannually during the dry season (November to April) and the wet season (May to October). Wild murine rodents were trapped using locally modified wire live traps measuring 12 cm (in width) × 28 cm (in length) × 12 cm (in height). These traps were baited with fresh corn. A total of 36 traps were strategically positioned in each sampling location according to a predefined grid line, with a set distance of 20 meters between each trap. Since murine rodents are nocturnal (9), the traps were deployed in the evening (between 3 and 6 PM) and checked for captures the following morning (between 5 and 8 AM). Animals other than murine rodents were released at the sampling location. Only the trapped murine rodent species were transported to the field stations for further investigation.

The rodents were euthanized through inhalation of an isoflurane overdose in a closed transparent chamber, following the ethical guideline established by Herbreteau et al. (34). Data including body weight, head-to-body length, ear length, hind foot length, tail length, the color of incisor, fur, and tail, and the number of mammae of female rodents were recorded to be used as a key for species identification (9). Initially, body weight and head-to-body length were used to classify specimens as rats or mice, followed by other parameters for accurate species identification. Genital appearance, as described by Herbreteau et al. (34), was used to determine sex and age class (juvenile or adult), with rodents showing underdeveloped genitalia classified as indeterminate. Murine rodent species were identified morphologically using biological measurements and identification keys (9, 35, 36). Subsequently, the rodents were dissected, and their gastrointestinal tracts were collected aseptically, preserved in 95% ethanol, and stored at 4°C until helminthological examination was conducted within 3 months.

### Helminthological examination and identification

Helminths were identified through the examination of the gastrointestinal tracts, with dissections conducted under a stereomicroscope (1.2X–1.4X) to provide detailed insights into their morphology. At this stage, the helminths were initially classified into nematodes, trematodes, and cestodes. For further taxonomic identification, the nematodes were cleared in lactophenol and mounted on temporary slides, while the cestodes and trematodes were stained with Semichon’s carmine. The morphological structure, including the mouthparts, tail features, and internal organs, was examined using a light microscope (4X–40X), and the species were identified based on established taxonomic keys (37–39). To obtain quantitative data, a comprehensive count of each helminth species within the individual murine hosts was conducted to assess the abundance of infection.

### Statistical analysis

The trap success (TS) rate served as a proxy for estimating the abundance of murine rodents, minimizing bias from unequal trap distributions across the habitats. The trap success rate was calculated using the following formula: Trap Success (TS) = $\frac{\mathrm{number}\;\mathrm{animal}\;\mathrm{caught}}{\mathrm{number}\;\mathrm{of}\;\mathrm{trap}\;\mathrm{efforts}}$ × 100 (9).

A chi-squared test was used to assess helminth infection prevalence across the habitats, seasons (dry vs. wet), groups of murine rodents (rat vs. mouse), sex (male vs. female), and age class (juvenile vs. adult). The Kruskal–Wallis test was used to evaluate the effect of habitat type on GI helminth abundance, while the Mann–Whitney U test was employed to compare the influence of age class, sex, groups of murine rodents, and seasons on GI helminths abundance.

The Chao and Jackknife indices were used to estimate true parasite species richness, addressing the under-sampling often observed in cryptic parasite communities. The Chao index predicts unobserved species based on the presence of rare species in the sample, while the Jackknife index estimates richness by systematically omitting parts of the dataset (40, 41). The Shannon index was used to quantify GI helminth species diversity, comparing the variations in abundance and species richness across the habitats and murine rodent species. All analyses were performed in Rstudio version 2024.04.2 + 764 “vegan” and “BiodiversityR” packages (42–44).

## Results

### Community structure of the murine rodents (trap success rate and species diversity)

A total of 380 murine rodents were trapped from 2,755 trap nights, yielding an overall trap success rate of 13.8%. Of the 380 trapped murine rodents, 59.7% (227/380) were male, while 39.7% (151/380) were female. The sex of two murine rodents could not be identified due to the underdevelopment of their genital organs. In terms of age class, 77.1% (293/380) were adults and the remaining were juveniles. Seasonality had an impact on the murine populations, with a higher number of trapped murine rodents and a higher trap success rate in the dry season compared to the wet season. Variation in the number of trapped murine rodents across the different types of habitats was observed. In addition, the forest habitat showed the highest number of trapped murine rodents (*n* = 124), while the solid waste sites revealed the highest trap success rate (17.6%), indicating the potential for high relative abundance of murine rodent populations in these two habitats. Details of the trapped murine rodents in this study are shown in Table 1.

**Table 1:** Number of trapped murine rodent hosts, trap success rate (%), prevalence of gastrointestinal helminth infection (%), mean abundance (MA), mean intensity (MI), parasite species richness (PSR) indices, and diversity index divided into types of habitat, season, age class, and sex.

	Total number of murine rodents	Trap success rate (%)	Prevalence of infection (%)	Mean Abundance (number of helminths per host ± SE)	Mean intensity (number of helminths per infected host ± SE)	Parasite Species Richness (PSR)			Diversity index (Shannon)
						Observed PSR	Estimated PSR (Chao)	Estimated PSR (Jackknife)	
Habitat									
Solid waste sites	102	17.6	74.5	35.5 ± 7.9	47.6 ± 10.2	12	13.98	13.98	1.38
Natural forests	124	12.8	90.3	40.6 ± 6.5	45.0 ± 7.1	14	14.49	14.99	1.63
Dense understory lands (DUL)	109	15.3	90.8	30.9 ± 4.9	34.0 ± 5.3	12	13.98	13.98	1.52
Sparse understory lands (SUL)	45	9.1	95.6	38.2 ± 12.8	39.9 ± 13.4	11	16.87	14.91	1.53
Season									
Wet	174	12.0	89.7	50.9 ± 7.1	56.8 ± 7.8	14	14.99	15.99	1.64
Dry	206	15.7	84.5	23.7 ± 4.3	28.1 ± 3.0	15	16.99	16.99	1.65
Age class^*^									
Adult	293	10.6	89.8	38.9 ± 4.2	43.3 ± 4.6	16	18.99	18.99	1.70
Juvenile	75	2.7	73.33	19.9 ± 4.1	27.1 ± 5.3	11	12.97	12.97	1.49
Sex^*^									
Male	227	8.3	90.31	45.1 ± 5.3	49.9 ± 5.7	15	16.99	16.91	1.66
Female	151	5.5	81.5	23.2 ± 4.4	28.5 ± 5.2	14	15.99	15.99	1.73
Murine species									
*Bandicota indica*	4	0.1	75.0	55.8 ± 35.2	74.3 ± 42.3	6	13.50	9.75	0.87
*Bandicota savilei*	5	0.2	100	42.0 ± 24.9	42.0 ± 24.9	6	12.40	9.20	0.38
*Maxomys surifer*	30	1.1	90.0	36.3 ± 8.1	40.4 ± 8.6	4	4.00	4.96	0.90
*Rattus rattus* complex	80	2.9	92.5	69.7 ± 13.9	75.4 ± 14.8	12	12.25	12.99	1.13
*Rattus exulans*	9	0.3	11.1	1.8 ± 1.8	16	1	1.00	1.89	NA
*Mus cervicolor*	214	7.8	86.0	25.5 ± 2.9	29.7 ± 3.3	13	13.25	13.99	1.41
*Mus caroli*	37	1.3	94.6	31.3 ± 7.6	33.1 ± 8.0	9	9.97	10.94	1.12
*Mus pahari*	1	0.1	100	1	1	1	1.00	1.00	NA

The solid waste sites had the highest trap success rate and the lowest prevalence of the infection, but the highest mean intensity. In contrast, the natural forests exhibited the highest species richness and diversity. *Mus cervicolor* was the dominant species captured, showing the highest parasite richness and diversity.

* Age could not be classified in 12 murine rodents, and sex could not be identified in two murine rodents.

**Figure 1:**
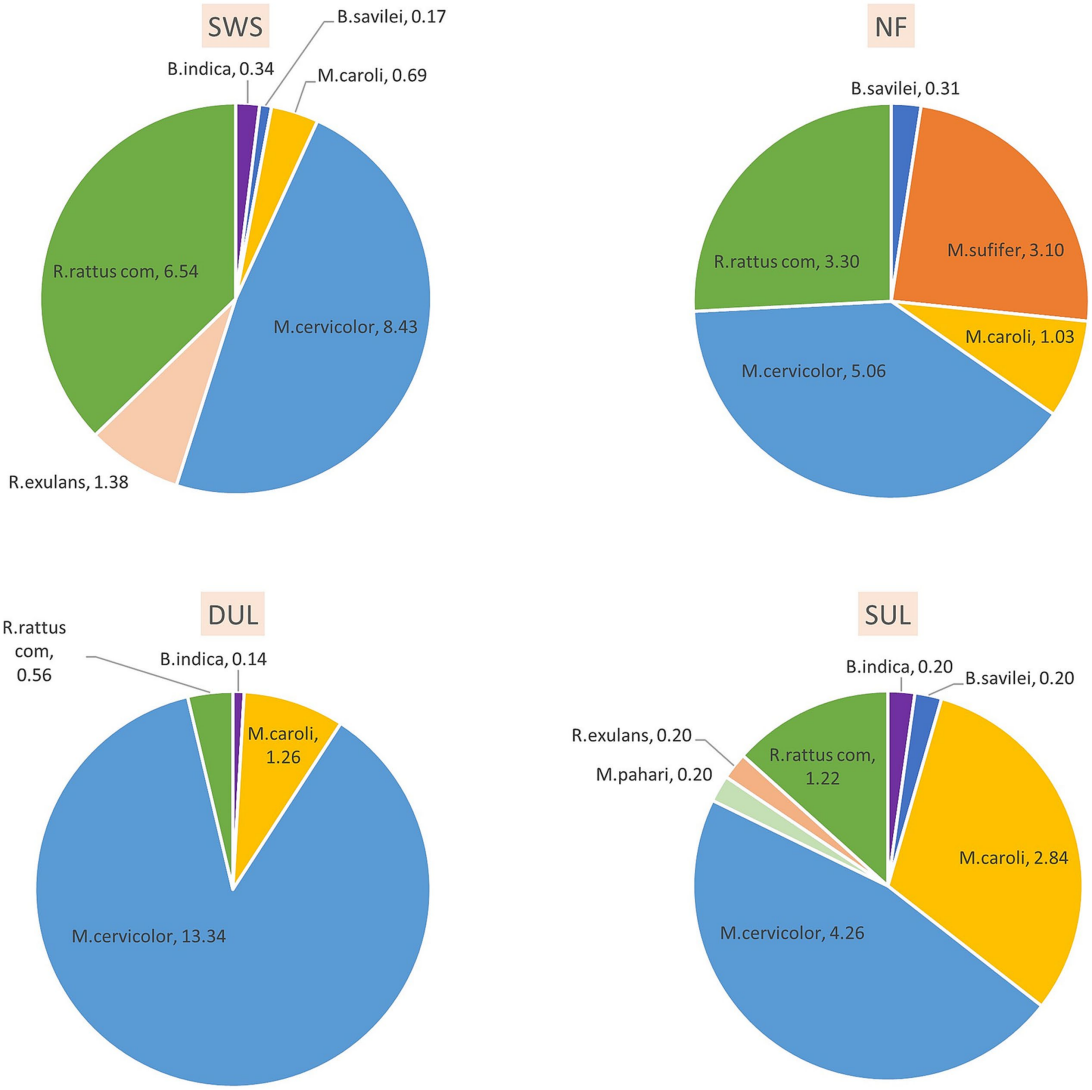
The composition of murine rodents varied across different habitats, reflecting the differing habitat suitability for each species. *Mus cervicolor* (the fawn-colored mouse) was dominant in all habitat types observed in this study, especially in the agricultural areas, while *Maxomys surifer* (the red spiny rat) was found exclusively in the forest habitat, where it had the second-highest abundance. SWS, solid waste sites; NF, natural forests; DUL, dense understory lands; SUL, sparse understory lands.

According to the morphological keys, eight murine species were identified. Among all trapped murine rodents, *Mus cervicolor* (*n* = 214) and *Rattus rattus* complex (*n* = 80) were the most abundant species, with trap success rates of 7.8 and 2.9, respectively. *Mus cervicolor* was the numerically dominant species in all habitat types, especially in the dense understory lands (DUL). In addition, the composition of the murine species varied in each type of the habitat. For example, the agricultural habitat types (DUL and SUL) showed a higher proportion (>75% of the total murine population) of *Mus* spp., including *M. cervicolor, M. caroli, and M. pahari,* compared to the other habitat types. On the other hand, *Maxomys surifer* was the second most abundant murine rodent species in the NF, while none were found in the other habitat types. In addition, a large proportion of *Rattus* spp. were found in the SWS. Details of the murine species composition in each type of the habitat are shown in Figure 1. The Shannon index was used to reveal the distinct murine diversity in the natural forests (1.35), SUL (1.32), SWS (1.17), and DUL (0.49), respectively.

### Prevalence of gastrointestinal helminth infection

Of the 380 trapped murine rodents, gastrointestinal helminths were found in 330, resulting in a prevalence of gastrointestinal helminth infection of 86.8%. An investigation into the relationship between the prevalence of gastrointestinal helminth infection and factors, (habitat type, season, age, sex, and murine species), revealed distinctive patterns. All habitats showed high prevalence of the infection, affecting more than 70% of the total population. The highest prevalence of the infection was observed in the SUL (95.56%), whereas the lowest prevalence was recorded in the SWS (74.51%). Habitat was the only exogenous factor that had a statistically significant relationship with the prevalence of the infection (*χ*^2^ = 19.394, *p* < 0.01). The prevalence of the infection was not significantly different (*χ*^2^ = 1.7919, *p* = 0.1807) between the seasons, although the prevalence of the infection in the wet season (89.66%) was slightly higher than that in the dry season (84.47%). The endogenous characteristics, including age Class (*χ*^2^ = 12.362, *p* < 0.01) and sex (*χ*^2^ = 5.4426, *p* = 0.01965), were found to affect the prevalence of the infection with statistical significance, with the adult and male murine rodents showing higher prevalence (Table 1).

### Abundance and intensity of the gastrointestinal helminths

In this study, a total of 13,740 individual gastrointestinal helminths were quantified from 380 trapped murine rodent hosts, resulting in a mean abundance (MA) of 36.2 helminths per host and a mean intensity (MI) of 41.6 helminths per host. The mean abundance and mean intensity of helminth infection varied by habitat, but there was no significant difference (Kruskal–Wallis chi-squared = 105.1, *p* = 0.344). In addition, the highest mean abundance was observed in the murine rodents from the natural forests (MA = 40.6 ± 6.5), while the SWS exhibited the highest mean intensity of helminth infection at 47.6 ± 10.2. Significant variations in gastrointestinal helminth abundance/intensity between the seasons were observed, with the wet season showing higher helminth abundance/intensity than the dry season (Mann–Whitney U test, W = 13,230, *p* < 0.01). Age, sex, and murine species also significantly impacted (*p* < 0.01) the abundance and intensity of gastrointestinal helminth infection in the murine rodents; see details in Table 1.

### Species richness and diversity of the gastrointestinal helminths

**Figure 2:**
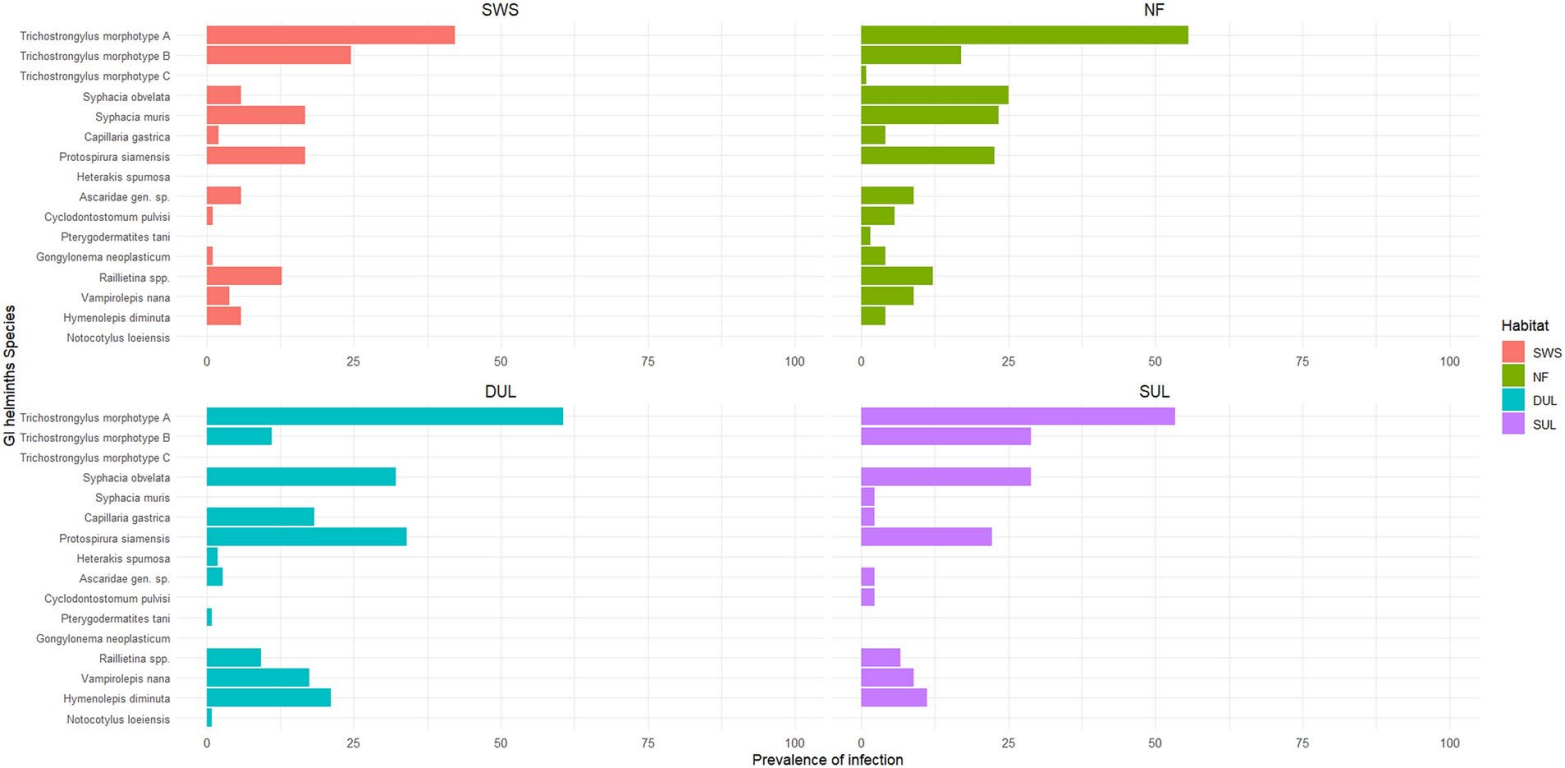
The prevalence of gastrointestinal helminth infections (%) varied across different habitat types. *Trichostrongylus* morphotype A was the most common helminth found in this study, while *Trichostrongylus* morphotype C was exclusively found in natural forests. The prevalence of the infection was also linked to the presence of specific host species, which varied by habitat. SWS, solid waste sites; NF, natural forests; DUL, dense understory lands; SUL, sparse understory lands.

A total of 16 species (or taxa) of the gastrointestinal helminths were morphologically identified in this study. *Trichostrongylidae* gen. sp. exhibited the highest population (*n* = 6,480), followed by *Syphacia muris* (*n* = 3,573) and *Syphacia obvelata* (*n* = 2,521). Based on the tail morphology, *Trichostrongylidae* gen. sp. was categorized into three morphotypes: morphotype A (*n* = 3,676), morphotype B (*n* = 2,796), and morphotype C (*n* = 8; Supplementary Figure S2). The total number and prevalence of infection of each helminth are shown in Figure 2 and Supplementary Table S1. Parasite species richness (PSR) was determined through microscopic examination, and the estimated true PSR was calculated using the Chao and Jackknife indices, revealing that the murine rodents living in the natural forest exhibited the highest PSR, with 14 identified species (or distinct taxa), followed by the murine rodents in the SWS and DUL, which hosted 12 species of GI helminths (Table 1). Of the 16 identified GI helminth species, 9 species were consistently found in every type of habitat, including *Ascaridae* gen. sp., *Protospirura siamensis*, *Syphacia obvelata*, *Capillaria gastrica*, *Trichostrongylus* morphotype A, Trichostrongylus morphotype B, *Hymenolepis diminuta*, *Vampirolepis nana*, and *Raillietina* spp. In contrast, *Notocotylus loeiensis* was only found in one murine rodent among the total of 380 murine rodents (Figure 2). GI helminth diversity was assessed using PSR, and the abundance across the habitats was determined using the Shannon–Wiener index, demonstrated varying values for each habitat: 1.63 for the natural forests, 1.52 for the dense understory lands, 1.53 for the sparse understory lands, and 1.38 for the solid waste sites.

### Zoonotic gastrointestinal helminths

Among the 16 species of gastrointestinal helminths investigated in this study, 6 were identified as zoonotic parasites. Notably, three of these parasites were cestodes, including *Raillietina* spp., *Hymenolepis diminuta*, and *Vampirolepis nana*, while the remaining three were nematodes, including *Syphacia obvelata*, *Syphacia muris,* and *Cyclodontostomum purvisi*. The prevalence of the infection for each zoonotic parasite was 10.8, 10.3, 10, 22.4, 12.4, and 2.4%, respectively. The prevalence of zoonotic helminth infection was notably higher and showed significant differences in the surrounding habitats compared to the SWS (χ^2^ = 24.638, *p* < 0.01; Table 2). The overall prevalence of the infection was quite similar between the seasons, with 56.3% in the wet season and 50.9% in the dry season (*χ*^2^ = 0.88102, *p* = 0.3479). Furthermore, a significantly higher prevalence of zoonotic helminth infection was observed in the adult (*χ*^2^ = 4.7984, *p* = 0.028) and male (*χ*^2^ = 15.643, *p* < 0.01) rodents (Table 2).

**Table 2:** Prevalence of the infection, total abundance, mean abundance, mean intensity, zoonotic parasite species richness (ZPSR), and parasite diversity of the zoonotic parasites found in this study, divided into types of habitat, season, age, class, sex, and murine group.

	Prevalence of zoonotic parasite (%)	Total abundance of zoonotic parasites	Mean Abundance (MA) of zoonotic parasite	Mean Intensity (MI) of zoonotic parasite	Observed ZPSR	Estimated ZPSR (Chao)	Estimated ZPSR (Jackknife)	Diversity index (Shannon)
Habitat								
Solid waste sites	33.3	1,731	17.0 ± 5.7	17 ± 5.7	6	6.00	6.99	0.53
Natural forests	62.9	2,827	22.8 ± 5.5	36.2 ± 8.4	6	6.00	6.00	0.82
Dense understory lands (DUL)	62.4	925	8.5 ± 2.6	13.6 ± 4.0	4	4.00	4.00	0.43
Sparse understory lands (SUL)	51.1	860	19.1 ± 11.6	37.4 ± 22.3	6	6.98	7.96	0.80
Season								
Wet	56.3	3,790	21.8 ± 5.7	38.7 ± 9.7	6	6.00	6.00	0.75
Dry	50.9	2,553	12.4 ± 2.1	24.3 ± 3.8	6	6.00	6.00	0.86
Age class								
Adult	56.3	4,843	16.5 ± 3.0	29.4 ± 5.3	6	6.00	6.00	0.88
Juvenile	41.3	789	10.5 ± 3.4	25.4 ± 7.6	6	6.49	6.99	0.69
Sex								
Male	61.7	4,713	20.8 ± 3.9	33.7 ± 6.0	6	6.00	6.00	0.83
Female	40.4	1,626	10.8 ± 4.1	26.7 ± 9.8	6	6.00	6.00	0.90
Murine group								
*Bandicota indica*	50.0	117	29.3 ± 28.9	58.5 ± 57.5	3	5.25	5.25	0.14
*Bandicota savilei*	40.0	5	1 ± 0.6	2.5 ± 0.5	3	5.40	5.40	1.05
*Maxomys surifer*	53.6	676	24.1 ± 6.9	45.1 ± 10.3	1	1.00	1.00	NA
*Rattus rattus* complex	67.1	2,274	28.8 ± 9.6	42.9 ± 13.9	5	5.00	5.99	0.18
*Rattus exulans*	11.1	16	1.8 ± 1.8	16	1	1.00	1.89	NA
*Mus cervicolor*	50.0	1,888	9.2 ± 1.8	18.3 ± 3.5	5	5.00	5.00	0.31
*Mus caroli*	54.3	655	18.7 ± 7.1	34.5 ± 12.0	4	4.97	5.95	0.11
*Mus pahari*	NA	NA	NA	NA	NA	NA	NA	NA

The natural forests posed a potential risk for zoonotic GI helminths, showing the highest prevalence of the infection and mean abundance, with relatively high parasite richness and diversity compared to the other habitats.

When only zoonotic parasites were considered, a total of 6,343 worms were quantified, with a mean abundance (MA) of 16.7 ± 2.8. The murine rodents from the natural forests (Kruskal–Wallis chi-squared = 77.711, *p* = 0.07318) demonstrated the highest mean abundance and mean intensity, with values of 22.8 and 36.2, respectively. The seasonal effect still showed higher mean abundance of zoonotic helminths in the wet season compared to the dry season (W = 17,379, *p* = 0.59). While the mice population (W = 14,275, *p* = 0.053) had 2,661 individual zoonotic parasites (MA = 10.6), the rat population exhibited 3,682 individual zoonotic parasites (MA = 28.8). Although these differences were not statistically significant, the patterns aligned with the overall gastrointestinal helminth prevalence trends, as detailed in Table 2. Zoonotic parasite species richness (ZPSR) was defined as the count of species, indicating that the natural forests, sparse understory lands, and solid waste sites exhibited the highest ZPSR, each hosting a total of six identified species. In contrast, the dense understory lands accommodated four parasite species each. Zoonotic helminth diversity, assessed using the Shannon–Wiener index, showed varying values, with the highest index found in the natural forests (Table 2).

## Discussion

This study was the first to assess the potential threats of GI helminths in murine rodents captured from solid waste sites and forest-adjacent areas in Nakhon Ratchasima Province, northeastern Thailand. A total of 380 murine rodents were trapped across four habitat types and examined for GI helminths using morphological keys. High trap success rates were observed in all habitats except the spare understory lands, indicating that this habitat is unsuitable for murine rodents due to the lack of hiding places at ground level (9, 45). On the contrary, the highest trap success rate was noted at the solid waste sites, indicating a potential breeding ground for murine rodents. In addition, the trap success rate was higher during the dry season, likely due to reduced food availability, which led the murine rodents to be more attracted to the bait (46).

Eight murine species were identified, with *Mus cervicolor* being the most predominantly trapped species in this study. This finding is consistent with those of previous studies (9, 34, 47). Its adaptability to various environments highlights its potential role in pathogen transmission (8, 48, 49). Moreover, *Mus cervicolor* was one of the two species carrying the zoonotic parasite, *Syphacia obvelata*, which showed high prevalence of infection in this study. This finding underscores the need for monitoring *S. obvelata* infection in humans within this area. Another notable finding was *Maxomys surifer*, which was exclusively found in the forested area, carrying four helminth species: *Syphacia muris* and three Trichostrongylus morphotypes (A, B, and C). The exclusive presence of this murine species in the forested area, in contrast to earlier research (50, 51), highlights a potential habitat shift due to human disturbances (52, 53), which can potentially facilitate parasite spillback or spillover into new hosts.

The overall prevalence of GI helminth infection in this study was 86.8%, which was notably higher than the prevalence reported from northern and northeastern Thailand, ranging from 55.1 to 71.54% (51, 54). One factor contributing to this higher prevalence is the inclusion of multiple murine rodent species in our study, compared to earlier research that focused on a single murine species. This broader sampling likely captured a wider range of host–parasite dynamics and interactions, increasing the observed prevalence. Seasonal differences also influenced the helminth dynamics, with higher infection prevalence and abundance observed during the wet season (55, 56), likely due to favorable environmental humidity for helminth eggs to hatch and infect hosts (26, 57). The differences in location, season, habitat characteristics, and trapping strategies could have influenced the variations in the prevalence of the infection (58, 59). These findings emphasize the importance of ecological factors in shaping parasite communities and highlight the value of examining diverse host populations to gain a better understanding of infection dynamics.

The factors associated with the prevalence and abundance of GI helminth infection included sex, age, and species. The adult male murine rodents exhibited higher prevalence and abundance of helminths, likely due to their larger body size, which can accommodate more helminths (51, 60–63). Similarly, the rats, with their larger body size, showed higher parasite abundance than the mice (64, 65). This suggests that habitats dominated by rats, such as solid waste sites and forests, may exhibit greater helminth burden. In-depth habitat analysis is further recommended to evaluate the ecological factors linked to the presence of murine rodents and their GI helminths.

Parasitic infections in murine rodents are potentially influenced by ecological factors, differing significantly between natural and human-modified habitats (58, 66). Natural forests provide stable ecosystems that support diverse parasite life cycles and interspecies interactions, which can regulate or promote parasite diversity and infection rates. This study observed moderate to high parasite prevalence and diversity in the murine rodents from the forest habitats compared to those from the solid waste sites. In contrast, human-modified habitats may disrupt parasite life cycles, particularly those requiring intermediate hosts, while favoring parasites with simpler life cycles that can adapt and thrive (66). In addition, changes in diet can influence parasite exposure; for instance, rodents in solid waste sites often forage on anthropogenic food sources, altering their exposure to helminth infective stages. Moreover, intensive agriculture and monoculture practices, often associated with high population of murine rodents, can facilitate parasite transmission by increasing contact between hosts (8, 67, 68), as observed in this study.

Among the 16 species of gastrointestinal helminths examined, *Trichostrongylus* morphotype A emerged as the predominant GI helminth, with high prevalence and abundance across all habitat types. In addition, both *Trichostrongylus* morphotypes A and B were consistently present in each habitat (Figure 2), consistent with the findings from previous studies (50, 51, 69, 70). Due to its direct life cycle and the adaptability of its larvae to various environmental conditions (71), it is therefore common to find this *Trichostrongylidae* gen. sp. in most areas where hosts are present. *Trichostrongylidae* gen. sp. is a gastrointestinal helminth in ruminants, rodents, pigs, horses, birds, and humans, with a worldwide distribution (39). Although *Trichostrongylidae* gen. sp. in rodents has not been reported as zoonotic, it can still affect the well-being of both host and non-host species, potentially causing symptoms such as mild abdominal discomfort and diarrhea (72, 73). In addition, due to its small size and morphological variation, individual identification of this parasitic taxon at the species level was not feasible. It is recommended that future research utilize molecular techniques for more accurate identification of these organisms. This approach will help elucidate its specific taxonomy (38, 74), examine host preferences, and assess potential impact on host populations.

Six zoonotic parasites were identified in this study, all previously documented as zoonotic in Thailand or other countries. While these parasites may remain asymptomatic at low infection levels, they can still cause illness in humans (75), presenting symptoms such as abdominal pain, vomiting, diarrhea, and malnutrition, particularly in children and immunocompromised individuals (26, 42, 76–84). Among them, *Syphacia* spp. are of particular concern as they are capable of infecting humans and causing abdominal pain and eosinophilia (75, 80). *Syphacia obvelata,* reported to be exclusively detected in mouse species (71), was the most prevalent zoonotic helminth observed in this study, with *Mus cervicolor* and *Mus caroli* identified as its primary hosts. Therefore, reducing specific murine rodent populations that serve as helminth reservoirs is considered a key strategy for minimizing the risk of human exposure to these zoonotic parasites (75). In addition, addressing public health concerns, improving sanitation and hygiene, and employing anthelmintic treatments are the recommended measures. To mitigate the issue of anthelmintic resistance, exploring herbal deworming as a sustainable alternative for parasite control could be a promising area for future research (85, 86).

This study found that approximately 8.5% of the murine rodents carried more than one hundred individual parasites without exhibiting visible clinical signs or disorders at the time of capture. This resilience may be attributed to the hosts’ robust immune response and overall health status. Within the gastrointestinal tract, cytokines such as IL-4 and IL-13 stimulate goblet cells, enhancing mucosal defense and reducing parasite-induced damage (87). Macrophages and eosinophils also play a pivotal role by secreting anti-inflammatory molecules that limit tissue damage and regulate the number of parasite species, contributing to the host ability to tolerate high parasite burden (88). These responses aim to contain parasites while minimizing harm to the host. Further research should delve deeper into the mechanisms underlying this balance, including investigations into blood parameters and species-specific immune responses.

In conclusion, the presence of murine rodents across all the habitat types, coupled with a high trap success rate, highlights their significant role in the transmission of zoonotic diseases, including the often-overlooked helminthiasis. The high prevalence of GI helminth infections, including six zoonotic species observed across the habitats, highlights the urgent need for comprehensive investigations into transmission dynamics. Solid waste sites, with the highest trap success rate, were identified as critical hotspots for multi-species interactions that may facilitate the spread of diseases. These sites also create an ideal environment for murine rodents due to abundant food sources, resulting in the highest number of trapped animals, thereby potentially amplifying the risk of disease transmission to humans and other species. To address this issue, it is essential to implement comprehensive waste management practices and establish effective monitoring programs to mitigate risks to public and veterinary health.

## Data Availability

The original contributions presented in the study are included in the article/Supplementary material, further inquiries can be directed to the corresponding author.

## Supplementary material

SUPPLEMENTARY FIGURE S1 Geographical location of each sampling locations deployed in this study. S1–S3 refered to three study sites that located near the Dong Phayayen-Khao Yai Forest Complex. Each study site consisted of at four different sampling locations including solid waste sites (SWS), natural forest (NF), dense understory lands (DUL), and sparse understory lands (SUL).

SUPPLEMENTARY FIGURE S2 The three Trichostrongyles morphotypes identified in this study were distinguished based on the structure of the caudal bulb at the tail structure. Morphotype A had a long and pointed caudal bulb, Morphotype B had a short and pointed caudal bulb, and Morphotype C had a short and blunt caudal bulb.

SUPPLEMENTARY TABLE S1 The prevalence of infection and total abundance of each gastrointestinal helminths found in this study.

## References

[1] Kaza S Yao LC Bhada-tata P van Woerden F. What a waste 2.0: A global snapshot of solid waste management to 2050. World Bank, Washington, DC. (2018). Available at: http://hdl.handle.net/10986/30317 (Accessed June 1, 2023).

[2] World Health Organization. Waste and human health: Evidence and needs, WHO meeting report. World Health Organization, Bonn. (2015).

[3] Katiyar M. Solid waste management. Int J Sci Eng Techno. (2016) 3:117–124. doi: 10.5958/2395-3381.2016.00015.0

[4] Vinti G Bauza V Clasen T Medlicott K Tudor T Zurbrügg C. Municipal solid waste management and adverse health outcomes: a systematic review. Int J Environ Res Public Health. (2021) 18:1–26. doi: 10.3390/ijerph18084331, PMID: 33921868 PMC8072713

[5] Katlam G Prasad S Aggarwal M Kumar R. Trash on the menu: patterns of animal visitation and foraging behaviour at garbage dumps. Curr Sci. (2018) 115:2322–6. doi: 10.18520/cs/v115/i12/2322-2326

[6] Krystosik A Njoroge G Odhiambo L Forsyth JE Mutuku F LaBeaud AD. Solid wastes provide breeding sites, burrows, and food for biological disease vectors, and urban zoonotic reservoirs: a call to action for solutions-based research. Front Public Health. (2020) 7:1–17. doi: 10.3389/fpubh.2019.00405, PMID: 32010659 PMC6979070

[7] Schroder GD Hulse M. Survey of rodent populations associated with an urban landfill. Am J Public Health. (1979) 69:713–5. doi: 10.2105/AJPH.69.7.713, PMID: 453401 PMC1619101

[8] Pitt WC Beasley J Witmer GW. Ecology, impacts, and management of invasive rodents in the United States. Ecology and management of terrestrial vertebrate invasive species in the United States. Boca Raton, FL: CRC Press (2017). 193–220.

[9] Aplin KP Brown PR Jacob J Krebs CJ Singleton GR. Field methods for rodent studies in Asia and the indo-Pacific. Australian Centre for International Agricultural Research (ACIAR): Canberra (2003).

[10] Douangboupha B Brown PR Khamphoukeo K Aplin KP Singleton GR. Population dynamics of rodent pest species in upland farming systems of Lao PDR. Kasetsart J (Nat Sci). (2009) 43:125–31.

[11] Morand S Bordes F Chen HW Claude J Cosson JF Ribas A. Global parasite and *Rattus* rodent invasions: the consequences for rodent-borne diseases. Integr Zool. (2015) 10:409–23. doi: 10.1111/1749-4877.12143, PMID: 26037785

[12] Villafañe IEG Cavia R Vadell MV Suárez OV Busch M. Differences in population parameters of *Rattus norvegicus* in urban and rural habitats of Central Argentina. Mammalia. (2013) 77:187–93. doi: 10.1515/mammalia-2012-0075, PMID: 39220592

[13] Yu H Jamieson A Hulme-Beaman A Conroy CJ Knight B Speller C. Palaeogenomic analysis of black rat (*Rattus rattus*) reveals multiple European introductions associated with human economic history. Nat Commun. (2002) 13:2399. doi: 10.1038/s41467-022-30009-z, PMID: 35504912 PMC9064997

[14] Claveria FG Causapin J de Guzman MA Toledo MG Salibay C. Parasite biodiversity in *Rattus* spp. caught in wet markets. Southeast Asian J Trop Med Public Health. (2005) 36 Suppl 4:146–8. PMID: 16438200

[15] Hwang K Chen E. Clinical studies on angiostrongyliasis Cantonese among children in Taiwan. Southeast Asian J Trop Med Public Health. (1991) 22:194–9. PMID: 1822885

[16] Kandi V Koka SS Bhoomigari MR. Hymenolepiasis in a pregnant woman: a case report of *Hymenolepis nana* infection. Cureus (2019) 11:1–e3815, doi: 10.7759/cureus.3810, PMID: , e3810 30868024 PMC6402731

[17] Mustapha T Daskum AM Majid RA Unyah NZ. A review on rodent-borne parasitic zoonosis: public health risk to humans. South Asian J Parasitol. (2019) 3:1–15.

[18] Otto GM Franklin CL Clifford CB. Biology and diseases of rats In: Fox JG Anderson LC Otto GM Pritchett-Corning KR Whary MT, editors. Laboratory animal medicine. 3rd ed. Cambridge, MA: Academic Press (2015). 151–207.

[19] Duh D Hasic S Buzan E. The impact of illegal waste sites on a transmission of zoonotic viruses. Virol J. (2017) 14:1–7. doi: 10.1186/s12985-017-0798-1, PMID: 28728557 PMC5520353

[20] Priyanto D Ningsih DP. Identification of endoparasites in rats of various habitats. Health Sci Indones. (2014) 5:49–53.

[21] Herbreteau V Bordes F Jittapalapong S Supputamongkol Y Morand S. Rodent-borne diseases in Thailand: targeting rodent carriers and risky habitats. Infect Ecol Epidemiol. (2012) 2:18637. doi: 10.3402/iee.v2i0.18637, PMID: 22957129 PMC3426326

[22] Hulin MS Quinn R. Wild and black rats In: Suckow MA Weisbroth SH Franklin CL, editors. The laboratory rat. 2nd ed. Cambridge, MA: Academic Press (2006). 865–82.

[23] Terashima M Suyanto A Tsuchiya K Moriwaki K Jin ML Suzuki H. Geographic variation of *Mus caroli* from east and Southeast Asia based on mitochondrial cytochrome b gene sequences. Mammal Study. (2003) 28:67–72. doi: 10.3106/mammalstudy.28.67

[24] Baker DG. Parasitic diseases In: Suckow MA Franklin CL Weisbroth SH, editors. The laboratory rat. 2nd ed. Cambridge, MA: Academic Press (2003). 453–78.

[25] Betterton C. The intestinal helminths of small mammals in the Malaysian tropical rain forest: patterns of parasitism with respect to host ecology. Int J Parasitol. (1979) 9:313–20. doi: 10.1016/0020-7519(79)90080-8

[26] Chaisiri K Siribat P Ribas A Morand S. Potentially zoonotic helminthiases of murid rodents from the indo-Chinese peninsula: impact of habitat and the risk of human infection. Vector Borne Zoonotic Dis. (2015) 15:73–85. doi: 10.1089/vbz.2014.1619, PMID: 25629783 PMC4307097

[27] Chenchittikul M Daengpium S Hasegawa M Itoh T Phanthumachinda B. A study of commensal rodents and shrews with reference to the parasites of medical importance in Chanthaburi Province, Thailand. Southeast Asian J Trop Med Public Health. (1983) 14:255–9. PMID: 6685345

[28] Lerdthusnee K Nigro J Monkanna T Leepitakrat W Leepitakrat S Insuan S. Surveys of rodent-borne disease in Thailand with a focus on scrub typhus assessment. Integr Zool. (2008) 3:267–73. doi: 10.1111/j.1749-4877.2008.00100.x, PMID: 21396076

[29] Namue C Wongsawad C. A survey of helminth infection in rats (*Rattus* spp) from Chiang Mai moat. Southeast Asian J Trop Med Public Health. (1997) 28 Suppl 1:179–83. PMID: 9656373

[30] Rahdar M Elham-Al-Sadat R Vazirianzadeh B Alborzi A. Study of internal parasites of rodents in Ahvaz, south-west of Iran. Jundishapur J Health Sci. (2016) 9:1–5. doi: 10.17795/jjhs-29067

[31] Ribas A Saijuntha W Agatsuma T Thongjun C Lamsan K Poonlaphdecha S. Helminths in rodents from wet markets in Thailand. Helminthologia. (2016) 53:326–30. doi: 10.1515/helmin-2016-0036

[32] Tijjani M Majid RA Abdullahi SA Unyah NZ. Detection of rodent-borne parasitic pathogens of wild rats in Serdang, Selangor, Malaysia: a potential threat to human health. Int J Parasitol. (2020) 11:174–82. doi: 10.1016/j.ijppaw.2020.01.008, PMID: 32099788 PMC7031134

[33] Vitta A Polseela R Nateeworanart S Tattiyapong M. Survey of *Angiostrongylus cantonensis* in rats and giant African land snails in Phitsanulok province, Thailand. Asian Pac J Trop Med. (2011) 4:597–9. doi: 10.1016/S1995-7645(11)60154-5, PMID: 21914534

[34] Herbreteau V Jittapalapong S Rerkamnuaychoke W Chaval Y Cosson JF Morand S. (2011). Protocols for field and laboratory rodent studies. Available at: http://www.ceropath.org/FichiersComplementaires/Herbreteau_Rodents_protocols_2011.pdf (Accessed June 1, 2023).

[35] Marshall JT. Family Muridae: rats and mice In: Lekagul B McNeely JA, editors. Mammals of Thailand. 2nd ed. Bangkok: Association for the conservation of wildlife (1998). 397–487.

[36] Waengsothorn S Kenthao A Latinne A Hugot JP. Rodents within the Centre for Thai national reference collections (CTNRC): past, present and future. Kasetsart J. (2009) 43:118–24.

[37] Anderson R Chabaud A Willmot S. Keys to the nematode parasites of vertebrates: Archival volume. Wallingford: CABI Publishing (2009).

[38] Taylor MA Coop RL Wall RL. Veterinary helminthology In: Veterinary parasitology. 4th ed. Chichester: Wiley-Blackwell (2015). 1–109.

[39] Schmidt GD Roberts LS Janovy J. Foundations of parasitology. 8th ed. Boston: McGraw-Hill Higher Education (2009).

[40] Magurran AE. Measuring biological diversity. J Torrey Bot Soc. (2004) 131:277–7. doi: 10.2307/4126959, PMID: 38532229

[41] Walther BA Morand S. Comparative performance of species richness estimation methods. Parasitology. (1998) 116:395–405. doi: 10.1017/S0031182097002230, PMID: 9585941

[42] Kindt R Coe R. BiodiversityR: a package for community ecology and suitability analysis. R package version 2.14-3. (2005). Available at: http://www.worldagroforestry.org/output/tree-diversity-analysis (Accessed May 15, 2023).

[43] Oksanen J Blanchet FG Friendly M Kindt R Legendre P McGlinn D. Vegan: community ecology package. R package version 2.4-3. (2017). Available at: https://CRAN.R-project.org/package=vegan (Accessed May 15, 2023).

[44] Bush AO Lafferty KD Lotz JM Shostak AW. Parasitology meets ecology on its own terms: Margolis et al. revisited. J Parasitol. (1997) 83:575–83. doi: 10.2307/3284227, PMID: 9267395

[45] Schweinfurth MK. The social life of Norway rats (*Rattus norvegicus*). eLife. (2020) 9:1–26. doi: 10.7554/eLife.54020, PMID: 32271713 PMC7145424

[46] Boonsong P Hongnark S Suasa-ard K Khoprasert Y Promkerd P Hamarit G. Rodent management in Thailand In: Singleton GR Hinds LA Leirs H Zhang Z, editors. Ecologically-based rodent management. Bruce: ACIAR (1999). 338–57.

[47] Kishimoto M Kato M Suzuki H. Morphological and molecular recharacterization of the rodent genus *Mus* from Nepal based on museum specimens. Mammal Study. (2021) 46:297–308. doi: 10.3106/ms2020-0065

[48] Pilosof S Fortuna MA Cosson JF Galan M Chaisiri K Ribas A. Host-parasite network structure is associated with community-level immunogenetic diversity. Nat Commun. (2014) 5:1–9. doi: 10.1038/ncomms6172, PMID: 25312328

[49] Shiels AB Pitt WC Sugihara RT Witmer GW. Biology and impacts of pacific island invasive species. 11. *Rattus rattus*, The black rat (Rodentia: Muridae). Pac Sci. (2014) 68:145–84. doi: 10.2984/68.2.1

[50] Chaisiri K Chaeychomsri W Siruntawineti J Ribas A Herbreteau V Morand S. Gastrointestinal helminth fauna in rodents from Loei province, Thailand. SWU Sci J. (2010) 26:111–26.

[51] Chaisiri K Chaeychomsri W Siruntawineti J Ribas A Herbreteau V Morand S. Diversity of gastrointestinal helminths among murid rodents from northern and northeastern Thailand. Southeast Asian J Trop Med Public Health. (2012) 43:21–8. PMID: 23082550

[52] Balakirev AE Abramov AV Rozhnov VV. The phytogeography of red spiny rats *Maxomys surifer* (Rodentia, Muridae) in Indochina with comments on taxonomy and description of new subspecies. Zool Stud. (2017) 56:1–19. doi: 10.6620/ZS.2017.56-06 PMC6517739 31966205

[53] Pimsai U Pearch MJ Satasook C Bumrungsri S Bates PJJ. Murine rodents (Rodentia: Murinae) of the Myanmar-Thai-Malaysian peninsula and Singapore: taxonomy, distribution, ecology, conservation status, and illustrated identification keys. Bonn Zool Bull. (2014) 63:15–114.

[54] Chaisiri K Herbreteau V Ribas A Morand S. A study of great bandicoot (*Bandicota indica*) and their gastrointestinal helminths from northern and northeastern Thailand. Adv Sci J. (2010) 10:163–71.

[55] Alvi MA Alshammari A Ali RMA Rashid I Saqib M Qamar W. 2023. Molecular characterization of *Hydatigera taeniaeformis* recovered from rats: an update from Pakistan. Pak Vet J. (2023) 43:601–5. doi: 10.29261/pakvetj/2023/049

[56] Alvi MA Li L Ohiolei JA Qamar W Saqib M Tayyab MH. *Hydatigera taeniaeformis* in urban rats (*Rattus rattus*) in Faisalabad, Pakistan. Infect Genet Evol. (2021) 92:104873. doi: 10.1016/j.meegid.2021.104873, PMID: 33905888

[57] O’Connor LJ Kahn LP Walkden-Brown SW. Moisture requirements for the free-living development of *Haemonchus contortus*: quantitative and temporal effects under conditions of low evaporation. Vet Parasitol. (2007) 150:128–38. doi: 10.1016/j.vetpar.2007.07.021, PMID: 17920198

[58] Archer CE Appleton CC Mukaratirwa S Lamb J Corrie SM. Endoparasites of public health importance recovered from rodents in the Durban metropolitan area, South Africa. S Afr J Infect Dis. (2017) 32:57–66. doi: 10.1080/23120053.2016.1262579, PMID: 39659294

[59] Mohd Zain SN Behnke JM Lewis JW. Helminth communities from two urban rat populations in Kuala Lumpur, Malaysia. Parasit Vectors. (2012) 5:47. doi: 10.1186/1756-3305-5-47, PMID: 22397763 PMC3364890

[60] Muñoz M Robles MR Milano F Navone G. Helminth infection levels on *Rattus rattus* (Rodentia: Muridae) from Corrientes city, Argentina. Mastozool Neotrop. (2018) 25:221–7. doi: 10.31687/saremMN.18.25.1.0.18

[61] Grandón-Ojeda A Moreno L Garcés-Tapia C Figueroa-Sandoval F Beltrán-Venegas J Serrano-Reyes J. Patterns of gastrointestinal helminth infections in *Rattus rattus*, *Rattus norvegicus*, and *Mus musculus* in Chile. Front Vet Sci. (2022) 9:1–9. doi: 10.3389/fvets.2022.929208, PMID: 35847649 PMC9277659

[62] Kataranovski M Mirkov I Belij S Popov A Petrović Z Gačić Z. Intestinal helminths infection of rats (*Rattus norvegicus*) in the Belgrade area (Serbia): the effect of sex, age and habitat. Parasite. (2011) 18:189–96. doi: 10.1051/parasite/2011182189, PMID: 21678796 PMC3671415

[63] Omondi C Ogolla FO Odhiambo C. Assessment of wild rodents endoparasites in Kirimiri forest in Embu County, Kenya. Int J Adv Res Pub. (2019) 4:31–7.

[64] Arneberg P. Host population density and body mass as determinants of species richness in parasite communities: comparative analyses of directly transmitted nematodes of mammals. Ecography. (2002) 25:88–94. doi: 10.1034/j.1600-0587.2002.250110.x

[65] Paladsing Y Boonsri K Saesim W Changsap B Thaenkham U Kosoltanapiwat N. Helminth fauna of small mammals from public parks and urban areas in Bangkok metropolitan with emphasis on community ecology of infection in synanthropic rodents. Parasitol Res. (2020) 119:3675–90. doi: 10.1007/s00436-020-06897-9, PMID: 33001253

[66] Cable J Barber I Boag B Ellison AR Morgan ER Murray K. Global change, parasite transmission and disease control: lessons from ecology. Philos Trans R Soc Lond Ser B Biol Sci. (2017) 372:20160088. doi: 10.1098/rstb.2016.0088, PMID: 28289256 PMC5352815

[67] Heroldova M Bryja J Zejda J Tkadlec E. Structure and diversity of small mammal communities in agriculture landscape. Agric Ecosyst Environ. (2007) 120:206–10. doi: 10.1016/j.agee.2006.09.007

[68] Singleton GR Lorica RP Htwe NM Stuart AM. Rodent management and cereal production in Asia: balancing food security and conservation. Pest Manag Sci. (2021) 77:4249–61. doi: 10.1002/ps.6462, PMID: 33949075

[69] Coomansingh C Pinckney RD Bhaiyat MI Chikweto A Bitner S Baffa A. Prevalence of endoparasites in wild rats in Grenada. West Indian Vet J. (2009) 9:17–21.

[70] Nursyazana MT Mohdzain SN Jeffery J. Biodiversity and macroparasitic distribution of the wild rat population of Carey Island, Klang. Trop Biomed. (2013) 30:199–210. PMID: 23959485

[71] Marchiondo AA Cruthers LR Reinemeyer CR. Nematoda In: Marchiondo AA Cruthers LR Fouries JJ, editors. Parasiticide screening: In vitro and in vivo tests with relevant parasite rearing and host infection/infestation methods, volume 2. Cambridge, MA: Academic Press (2019). 135–355.

[72] Farrar J Garcia P Hotez P Junghanss T Kang G Lalloo D. Soil-transmitted helminths (Geohelminths) In: Manson's tropical diseases. 24th ed. Amsterdam: Elsevier. (2024). 772–96.

[73] Gutierrez Y. Other tissue nematode infections In: Tropical infectious diseases: Principles, pathogens, & practice, vol. 2. 2nd ed. Philadelphia: Saunders. (2006). 1231–47.

[74] Gibbons LM Khalil LF. A key for the identification of genera of the nematode family Trichostrongylidae Leiper, 1912. J Helminthol. (1982) 56:185–233. doi: 10.1017/S0022149X00034581, PMID: 7175163

[75] King CH. Helminthiasis epidemiology and control: scoring successes and meeting the remaining challenges. in Adv Parasitol. ed. Keiser N. J. Cambridge, MA: Academic Press. (2019) 103:11–30., PMID: 10.1016/bs.apar.2018.08.001 30878055

[76] Hasegawa H Syafruddin. *Cyclodontostomum purvisi* (Syn. *Ancistronema coronatum*) (Nematoda: Strongyloidea: Chabertiidae) from rats of Kalimantan and Sulawesi, Indonesia. J. Parasitol. (1994) 80:657–60. doi: 10.2307/3283208, PMID: 8064538

[77] Bhaibulaya M Indrangarm S. Man, an accidental host of *Cyclodontostomum purvisi* (Adams, 1933), and the occurrence in rats in Thailand. Southeast Asian J Trop Med Public Health. (1975) 6:391–4. PMID: 1221507

[78] Bogitsh BJ Carter CE Oeltmann TN. Intestinal tapeworms In: Bogitsh BJ Carter CE Oeltmann TN, editors. Human parasitology. 1st editor ed. Cambridge, MA: Academic Press (2013). 237–49.

[79] Jarošová J Antolová D Zalesny G Halán M. Oxyurid nematodes of pet rodents in Slovakia- a neglected zoonotic threat. Braz J Vet Parasitol. (2020) 29:e014319. doi: 10.1590/s1984-29612019072, PMID: 31576975

[80] Sapp SGH Bradbury RS. The forgotten exotic tapeworms: a review of uncommon zoonotic Cyclophyllidea. Parasitolology. (2020) 147:533–58. doi: 10.1017/S003118202000013X, PMID: 32048575 PMC7174715

[81] Sinniah B Sinniah D Singh M Poon GK. Prevalence of parasitic infections in Malaysian oil palm estate workers. Southeast Asian J Trop Med Public Health. (1978) 9:272–6. PMID: 725660

[82] Sirivichayakul C Radomyos P Praevanit R Pojjaroen-Anant C Wisetsing P. *Hymenolepis nana* infection in Thai children. J Med Assoc Thail. (2000) 83:1035–8. PMID: 11075970

[83] Stone WB. Potential helminth infections in humans from pet or laboratory mice and hamsters. Public Health Rep. (1966) 81:647–53. doi: 10.2307/4592796 19316507 PMC1919826

[84] William RA. A mouse oxyurid, *Syphacia obvelata*, as a parasite of man. J Parasitol. (1919) 6:89–93. doi: 10.2307/3270899, PMID: 38532229

[85] Qamar W Alkheraije KA. Anthelmintic resistance in *Haemonchus contortus* of sheep and goats from Asia–a review of in vitro and in vivo studies. Pak Vet J. (2023) 43:376–87. doi: 10.29261/pakvetj/2023.088

[86] Al-Saeed FA Bamarni SSI Iqbal KJ Rehman TU Faruk AZ Mahmood S. In vitro anthelmintic efficacy of *Haloxylon salicornicum* leaves extract using adult *Heamonchus contortus* worms. Pak Vet J. (2023) 43:91–6. doi: 10.29261/pakvetj/2022.091, PMID: 39639209

[87] Rückerl D. Characterizing activation, proliferation, and ontogeny of murine macrophages in parasitic helminth infections. Methods Mol Biol. (2018) 2018:225–41. doi: 10.1007/978-1-4939-7837-3-21 29761403

[88] Vacca F Le Gros G. Tissue-specific immunity in helminth infections. Mucosal Immunol. (2022) 15:1212–23. doi: 10.1038/s41385-022-00531-w, PMID: 35680972 PMC9178325

