# Supplementary figures and images for: Murine-related helminthiasis: a public health concern at solid waste sites around forest- adjacent communities in Thailand
Source: Front Vet Sci. 2025 Jan 15;11:1463046. doi: 10.3389/fvets.2024.1463046 (PMC11774862; doi:10.3389/fvets.2024.1463046)

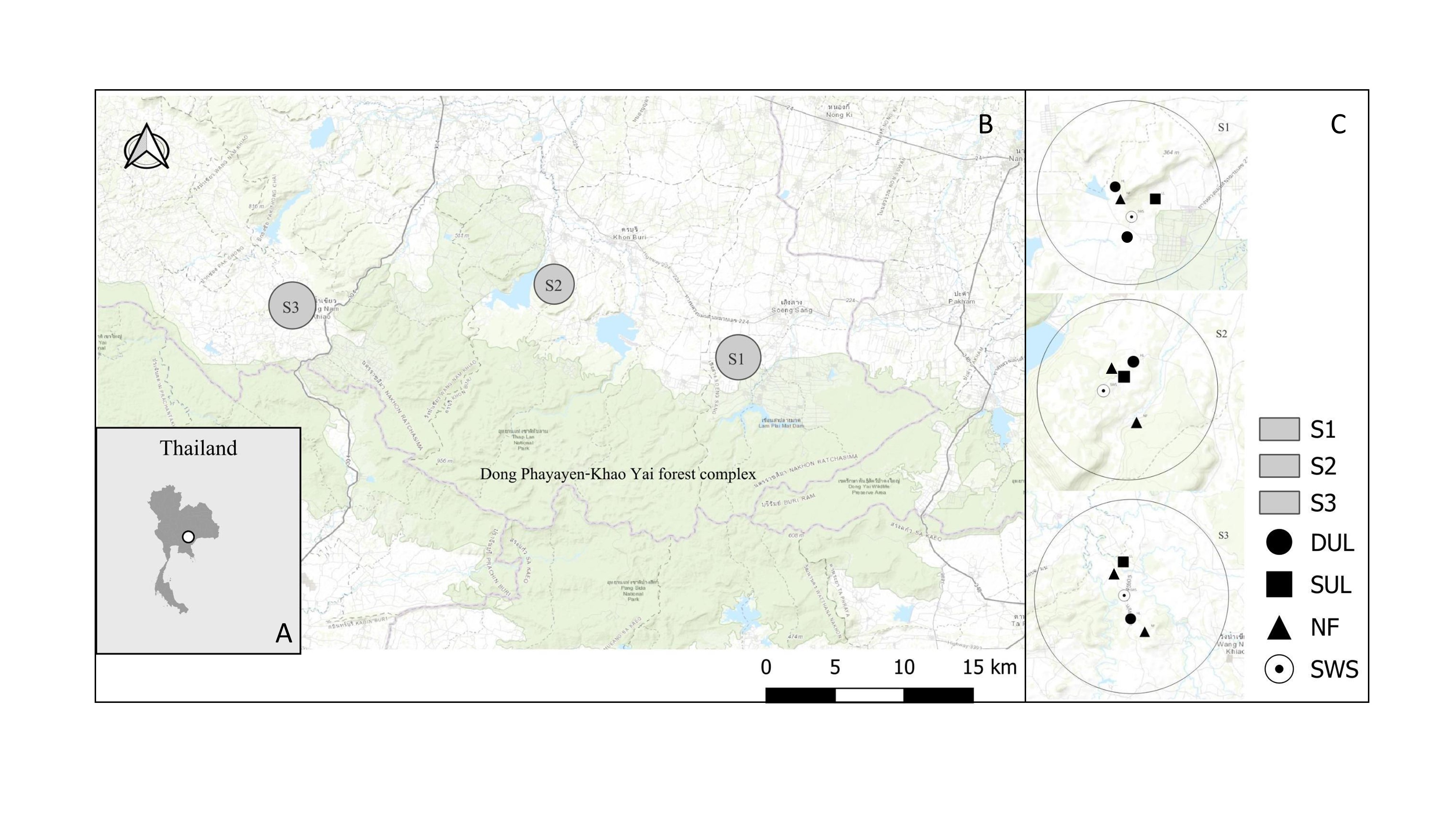

Supplement: SUPPLEMENTARY FIGURE S1 — Geographical location of each sampling locations deployed in this study. S1–S3 refered to three study sites that located near the Dong Phayayen-Khao Yai Forest Complex. Each study site consisted of at four different sampling locations including solid waste sites (SWS), natural forest (NF), dense understory lands (DUL), and sparse understory lands (SUL). [file Image_1.JPEG]

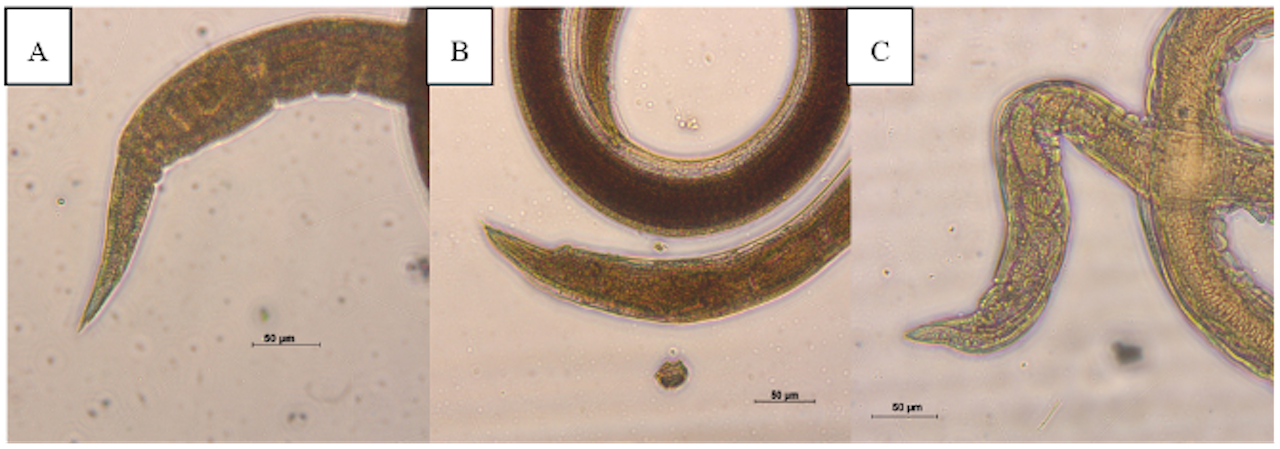

Supplement: SUPPLEMENTARY FIGURE S2 — The three Trichostrongyles morphotypes identified in this study were distinguished based on the structure of the caudal bulb at the tail structure. Morphotype A had a long and pointed caudal bulb, Morphotype B had a short and pointed caudal bulb, and Morphotype C had a short and blunt caudal bulb. [file Image_2.PNG]
