# Supplementary material for: Murine-related helminthiasis: a public health concern at solid waste sites around forest- adjacent communities in Thailand
Source: Front Vet Sci. 2025 Jan 15;11:1463046. doi: 10.3389/fvets.2024.1463046 (PMC11774862; doi:10.3389/fvets.2024.1463046)
Supplement: SUPPLEMENTARY TABLE S1 — The prevalence of infection and total abundance of each gastrointestinal helminths found in this study. [file Table_1.docx]

**Table S1** The prevalence of infection and total abundance of each gastrointestinal helminths found in this study.

| **Helminth species** | **Prevalence of infection**  **(%)** | **Total abundance  (Total parasite count)** |
| --- | --- | --- |
| **Nematode** |  |  |
| *Trichostroingylus* morphotype A | 53.2 | 3676 |
| *Trichostrongylus* morphotype B | 18.7 | 2796 |
| *Trichostrongylus* morphotype C | 0.3 | 8 |
| *Syphacia obvelata* | 22.4 | 2521 |
| *Syphacia muris* | 12.4 | 3573 |
| *Capillaria gastrica* | 7.4 | 162 |
| *Protospirura siamensis* | 24.2 | 610 |
| *Heterakis spumosa* | 0.5 | 3 |
| Ascaridae gen. sp. | 5.5 | 94 |
| *Cyclodontostomum purvisi* | 2.4 | 17 |
| *Pterygodermatites tani* | 0.8 | 13 |
| *Gongylonema neoplasticum* | 1.6 | 9 |
| **Cestode** |  |  |
| *Raillietina* spp. | 10.8 | 87 |
| *Vampirolepis nana* | 10.0 | 64 |
| *Hymenolepis diminuta* | 10.3 | 81 |
| **Trematode** |  |  |
| *Notocotylus loeiensis* | 0.3 | 26 |
